# A ‘benign’ extrapyramidal side effect masking a life-threatening neuroleptic malignant syndrome

**DOI:** 10.12669/pjms.36.6.2963

**Published:** 2020

**Authors:** Reinhard Dolp, Moin Ahmed Ansari, Michael Chan, Tariq Hassan

**Affiliations:** 1 Dr. Reinhard Dolp, Resident Physician, Postgraduate year 3, Department of Psychiatry, Queen’s University, Kingston, Ontario, Canada; 2 Dr. Moin Ahmed Ansari, FCPS. Head of the Department of Psychiatry, Liaqat University of Medical & Health Specialities, Hyderabad, Pakistan; 3 Dr. Michael Chan, FRCPC. Assistant Professor, Department of Psychiatry, Queen’s University, Kingston, Ontario, Canada; 4 Dr. Tariq Hassan, FRCPC (Forensic Psychiatry). Associate Professor, Head of Forensic Psychiatry, Department of Psychiatry, Queen’s University, Kingston, Ontario, Canada

**Keywords:** Neuroleptic malignant syndrome, Neuroleptics, Antipsychotics, Extra-pyramidal side effects, EPSE, NMS

## Abstract

The neuroleptic malignant syndrome is a rare, life-threatening idiosyncratic reaction to neuroleptic medication. The use of newer antipsychotics combined with its rare incidence has made NMS seem as a complication of the past. Here we report a patient in his early 20s suffering from a psychotic disorder developing a life-threatening neuroleptic malignant syndrome on an inpatient psychiatric ward in Canada without the characteristic overt change in autonomic stability. We review the clinical characteristics to facilitate the early recognition of neuroleptic malignant syndromes and discuss why this condition still is highly relevant for practising physicians.

## INTRODUCTION

Neuroleptic malignant Syndrome (NMS) is a psychiatric emergency with a mortality rate between 10 and 20%. Characteristically it presents with hyperpyrexia, muscle rigidity, dysautonomia, and mental status changes after the administration of a high-potency neuroleptic agents.^1^ Although the incidence of NMS with patients taking antipsychotics is less than 3%, it is of paramount importance to recognize it as early as possible to avoid potential lethal consequences such as kidney failure due to rhabdomyolysis or arrhythmias.^2^,^3^ In this case report, we describe a case that illustrates the complexities of recognizing and treating early indicators of NMS before it reaches all the international consensus diagnostic criteria for NMS.^4^ We will further discuss the challenges that arise once NMS is diagnosed with certainty.

## CASE REPORT

Our patient is a Caucasian male in his early 20s with a history of schizophrenia spectrum and substance use disorder. He has been experiencing auditory hallucinations and paranoid delusions since his early teens and had started consuming oral stimulants in form of crystal methamphetamine on a monthly basis in his late teens. We received this patient in our forensic psychiatry inpatient ward due to his aggressive and erratic behaviour in prison - punching walls and threatening inmates as well as family members. Apart from his mental illness, the patient had no history of any physical illness.

At the time of admission, he was on an oral antipsychotic, ziprasidone which was started several weeks before his imprisonment. Within two weeks, his affect became more restricted and he stopped taking his medications for five days due to a subjective lack of benefit. We could convince him to restart ziprasidone, but on a reduced dose. Within one month after admission, his behaviour progressively worsened in terms of angry spells and he refused to take his medications again. We collaboratively switched to a different oral antipsychotic, aripiprazole since he had felt best on this medication in the past and added low-dose oral clonazepam. The patient denied adverse side effects on the daily rounds. However, his irritability started to increase again shortly after starting aripiprazole. When asked, the patient insisted that his irritability is related to the new environment and that the aripiprazole helped him to stay calm. He insisted in a further dosage increase, which resulted in a significantly reduced irritability and anger spells after a few days.

Two weeks after increasing aripiprazole, the patient was wearing a thick pullover on the daily round despite warm temperature and was sweating. He was not agitated, or hypervigilant. He refused to talk but communicated with head shaking or nodding. Afraid of over-sedation, we reduced his clonazepam. The following two days he developed overtly unusual behaviour and bizarre persecutory delusions which had not been part of his previous symptoms. He started to constantly remove most of his clothing, was pacing the hallway, and unable to follow rules.

Due to non-compliance, increasing paranoia and violent behavior, we opted for a short-acting intramuscular first generation antipsychotic zuclopenthixol. The next day he was calmer and more compliant but showed a further increase in his paranoia with a new onset of vague suicidal thoughts. His movements presented slowed and his thinking disordered.

We diagnosed a psychotic state with some catatonic-like features without meeting the full criteria for catatonia. During the following night, the patient had some posture abnormalities in form of ambitendency and very mild posturing. His vitals were at baseline apart from mild tachycardia (135bpm). Physical examination revealed mild rigidity on arm extension. To rule out neuroleptic malignant syndrome, we took blood samples to determine creatinine kinase (CK). CK was elevated at >160,000 units/litre (norm: <200 u/L). Based on the clinical picture and the elevated CK, the diagnosis of beginning NMS was made.

The patient was immediately transferred to the ICU, where emergency treatment with dantrolene and bromocriptine was initiated. On the same day, the patient developed acute kidney injury which required dialysis. After several weeks in the ICU the patient’s kidney function recovered and he was transferred back to the forensic psychiatry unit.

## DISCUSSION

Our patient developed life-threatening rhabdomyolysis - one of the most feared complications of NMS. Even though, he did not meet the full criteria of NMS (hyperpyrexia, muscle rigidity, dysautonomia, and mental status changes) 4 at the time of diagnosis, muscle tissue breakdown at such a high level (CK>160,000 u/L) can’t be accounted by any other condition than by NMS. Due to the high risk of organ damage, urgent ICU admission and treatment of NMS was warranted.

**Table:** Teaching points

1	In clinical practice NMS does not present as a clear and easily distinguishable syndrome.
2	Recognition and treatment of NMS needs to be immediate and as early as possible, even before reaching all diagnostic criteria to prevent mortality and morbidity.
3	NMS always needs to be kept in mind after administering a high potency antipsychotic when developing signs of rigidity regardless of autonomic instability.
4	Creatinine kinase is an inexpensive test and should be ordered sooner rather than later.
5	Differential diagnosis to NMS after ruling out medical causes: acute psychosis, severe depression, serotonin syndrome (SS), malignant hyperthermia, and malignant catatonia.

In clinical practice NMS does not present as a clear and easily distinguishable syndrome. Our patient showed normal temperature, his muscle rigidity was minor and solely present in his arms. The only sign of dysautonomia was intermittent sweating and mild tachycardia which is not unusual for BMI >40kg/m^2^. His mental status change (irritability, refusal to talk) was not clearly attributable to the administration of a neuroleptic agent.

Given this history and the fact that we switched recently his antipsychotic medication, we attributed his change in behavior to a primarily psychotic state with catatonia-like features. Therefore, we decided to administer the zuclopenthixol - first as a shorter-acting IM injection (Clopixol-Acuphase®) to acutely stabilize the patient, with the option to continue with a long-acting IM preparation (zuclopenthixol decanoate). He did improve overnight and presented calmer and more compliant the following day indicating that diagnosis and treatment of worsening psychosis was correct and effective. By the time he developed more pronounced catatonic symptoms indicating NMS, he already had rhabdomyolysis and needed emergency treatment in the ICU.

This case demonstrates, how important it is to recognize even faint symptoms of NMS early to save the patient’s life, and, how difficult it is to distinguish NMS symptoms from other differential diagnosis. After the exclusion of medical reasons for movement abnormalities, mutism, and psychosis such as electrolyte disturbances, seizures, withdrawal, or porphyria, one has to consider acute psychosis, severe depression, serotonin syndrome (SS), malignant hyperthermia, and malignant catatonia.

The most commonly diagnosed related disorder is Serotonin Syndrome (SS).^5^ Frank SS appears most likely with hyperreflexia, shivering, myoclonus and GI symptoms compared to the hyperreflexia and rigidity of NMS. However, both syndromes overlap and can be hard to distinguish, especially in patients that receive selective serotonin reuptake inhibitors (SSRIs) and neuroleptics at the same time. In the here presented patient, no serotonergic drugs were used and SS could be excluded as a differential diagnosis. Malignant hyperthermia can present with the same symptoms as NMS but is usually triggered by volatile anesthetics or succinylcholine. In addition, it tends to have a more fulminant onset.^6^

The most difficult, but also the most important differential diagnosis is malignant/lethal catatonia.^6^ Both, NMS and malignant catatonia, can present with the same symptom complex of rigidity, altered mental status, and akinesia. Malignant catatonia usually has a more insidious onset with weeks of preceding negative symptoms such as apathy and social withdrawal. However, the distinction between those two life-threatening entities can be difficult but is of paramount importance: Malignant catatonia requires immediate administration of neuroleptics to save the patient’s life, whereas NMS requires the exact opposite to save the patient - immediate cessation of all anti-dopaminergic drugs.

At the highest risk for developing NMS are patients treated with “typical” (“1^st^ generation”) high-potency antipsychotics such as haloperidol or fluphenazine. However, every medication influencing central dopamine receptors can cause NMS - from “atypical” (“2^nd^ generation” and “3^rd^ generation”) antipsychotics to antiemetics like metoclopramide. NMS can even develop after cessation of dopaminergic drugs such as L-Dopa or bromocriptine – frequently used in the treatment of Parkinson’s Disease. It is important to know that NMS is not dose-dependent, it rather is an idiosyncratic reaction; Meaning it can occur even after the first dose of a causative agent. The risk is higher, however, if this medication is given in high dosages or parenteral.^7^

The most important step after identifying NMS is the immediate cessation of all neuroleptic or other dopamine receptor blocking agents, followed by supportive medical treatment. Aggressive hydration is warranted to reduce the risk of kidney failure due to rhabdomyolysis. The patient needs to be monitored for potential disseminated intravascular coagulation. Hyperpyrexia may require external cooling. Specific medical therapy has low evidence and is based on case reports only due to the low incidence of NMS with dantrolene, bromocriptine, and amantadine being the most commonly used agents; Dantrolene IV to relax the muscles, bromocriptine or amantadine orally to revert the dopamine receptor blockade.^6^,^8^

### Authors’ Contribution

**TH and MC:** Managed the patient whose case report this is and reviewed manuscript’s accuracy and flow.

**RD:** Prepared the manuscript and collected the patient chart data for the purpose of the case study

**MA:** Reviewed the manuscript and gave important recommendations in its preparation.

**TH** takes the responsibility and is accountable for all aspects of the work in ensuring that questions related to the accuracy or integrity of any part of the work are appropriately investigated and resolved.
